# 4-(*N*-Propan-2-ylcarbamo­yl)pyridinium perchlorate

**DOI:** 10.1107/S1600536810019306

**Published:** 2010-05-26

**Authors:** Bo Wang

**Affiliations:** a Ordered Matter Science Research Center, Southeast University, Nanjing 210096, People’s Republic of China

## Abstract

In the title compound, C_9_H_13_N_2_O^+^·ClO_4_^−^, the dihedral angle between the planes of the amide group and the pyridinium fragment is 34.11 (14)°. In the crystal, the cations are connected by N—H⋯O hydrogen bonds between the amide groups into chains extended along the *a* axis. Hydrogen bonds between the pyridinium N—H group and the perchlorate anions organize the chains into a two-dimensional network.

## Related literature

For the physical properties of simple mol­ecular–ionic crystals, see: Czupiński *et al.*, 2002 ▶; Katrusiak & Szafrański (1999 ▶, 2006 ▶). For related structures, see: Gholivand *et al.* (2007 ▶); Chen (2009 ▶); Zhang *et al.* (2009 ▶).
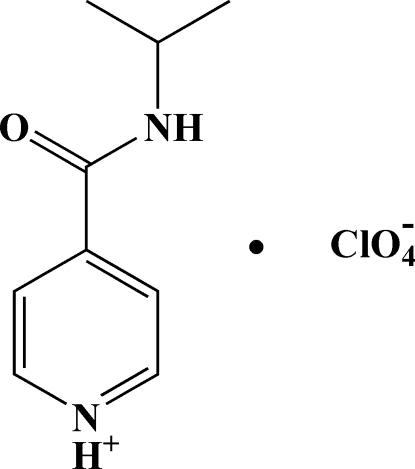


## Experimental

### 

#### Crystal data

C_9_H_13_N_2_O^+^·ClO_4_^−^
*M* *_r_* = 264.66
Triclinic, 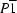

*a* = 4.9342 (3) Å
*b* = 8.973 (4) Å
*c* = 13.715 (10) Å
α = 93.046 (12)°
β = 91.07 (2)°
γ = 101.01 (3)°
*V* = 594.9 (5) Å^3^
*Z* = 2
Mo *K*α radiation
μ = 0.33 mm^−1^
*T* = 298 K
0.2 × 0.2 × 0.2 mm

#### Data collection

Rigaku SCXmini diffractometer
Absorption correction: multi-scan (*CrystalClear*; Rigaku, 2005 ▶) *T*_min_ = 0.887, *T*_max_ = 1.000
6065 measured reflections
2708 independent reflections
2160 reflections with *I* > 2σ(*I*)
*R*_int_ = 0.024

#### Refinement

*R*[*F* ^2^ > 2σ(*F* ^2^)] = 0.055
*wR*(*F* ^2^) = 0.159
*S* = 1.07
2708 reflections
154 parameters
H-atom parameters constrained
Δρ_max_ = 0.78 e Å^−3^
Δρ_min_ = −0.58 e Å^−3^

### 

Data collection: *CrystalClear* (Rigaku, 2005 ▶); cell refinement: *CrystalClear*; data reduction: *CrystalClear*; program(s) used to solve structure: *SHELXS97* (Sheldrick, 2008 ▶); program(s) used to refine structure: *SHELXL97* (Sheldrick, 2008 ▶); molecular graphics: *SHELXTL* (Sheldrick, 2008 ▶); software used to prepare material for publication: *PRPKAPPA* (Ferguson, 1999 ▶).

## Supplementary Material

Crystal structure: contains datablocks I, global. DOI: 10.1107/S1600536810019306/gk2274sup1.cif

Structure factors: contains datablocks I. DOI: 10.1107/S1600536810019306/gk2274Isup2.hkl

Additional supplementary materials:  crystallographic information; 3D view; checkCIF report

## Figures and Tables

**Table 1:** Hydrogen-bond geometry (Å, °)

*D*—H⋯*A*	*D*—H	H⋯*A*	*D*⋯*A*	*D*—H⋯*A*
N1—H1*B*⋯O5^i^	0.86	2.20	2.879 (3)	136
N1—H1*B*⋯O2^ii^	0.86	2.36	3.032 (4)	135
N2—H2*B*⋯O1^iii^	0.86	2.18	2.957 (3)	150

Symmetry codes: (i) 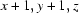; (ii) 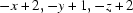; (iii) 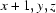.

**Fig. 1.**
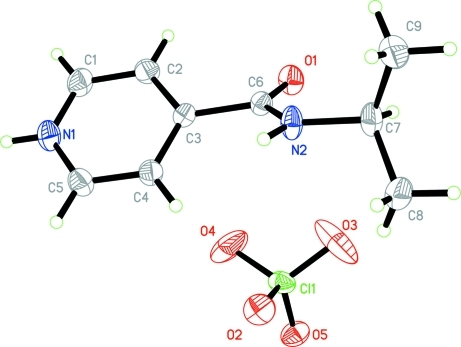
Molecular structure of the title compound, with displacement ellipsoids drawn at the 30% probability level.

**Fig. 2.**
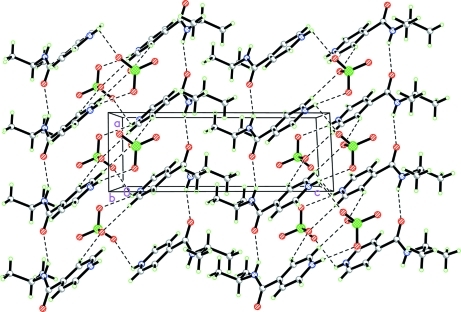
Crystal packing of the title compound viewed along the b axis showing the N—H···O and, C—H···O interactions (dotted line).

## supplementary crystallographic information

### Comment

Recently much attention has been devoted to simple molecular–ionic crystals containing organic cations and anions due to the tunability of their special structural features and their interesting physical properties (Czupiński *et al.*, 2002; Katrusiak & Szafrański, 1999; Katrusiak & Szafrański, 2006). For similar structures, see: Gholivand *et al.*, 2007; Chen, 2009. In our laboratory, the compound containing 4-(propan-2-ylcarbamoyl)pyridinium cation and ClO_4_^-^anion has been synthesized and its crystal structure is reported herein.

The asymmetric unit of the title compound, C_9_H_13_N_2_O^+^.ClO_4_^-^, consists of a 4-(propan-2-ylcarbamoyl)pyridinium cation and a ClO_4_^-^ anion (Fig 1). In the anion, the average Cl—O bond distances and O—Cl—O bond angles are 1.425 Å and 109.4°, respectively, confirming a tetrahedral configuration. In the 4-(propan-2-ylcarbamoyl)pyridinium cation , the pyridine N atom is protonated. In the cation,  the acyl group is twisted relative to the pyridine by 34.11(0.14)°. The torsion angle O1-C6-N2-C7 isof -2.7 (4)°. It shows that the four atoms are nearly coplanar.

Hydrogen bonds N—H···O and C—H···O make great contribution to the stability of the crystal structure (Table 1). The cations are connected by N—H···O hydrogen bonds between the amide groups into chains extended along the *a* axis. Hydrogen bonds between pyridinium N-H group and the perchlorate anions organize the chains into two-dimensional polymeric structure (Fig 2).

### Experimental

4-(Propan-2-ylcarbamoyl) pyridine (0.492 g, 3 mmol) (Zhang *et al.*, 2009), and HClO_4_ (0.42 g, 70%) were dissolved in 15 ml of ethanol. Single crystals of the title compound suitable for X-ray analysis were obtained on slow evaporation of the solvent over a period of 7 days.

The dielectric constant of title compound as a function of temperature indicates that the permittivity is basically temperature-independent (ε=C/ (T—T_0_)), suggesting that this compound should not be a real ferroelectric and that no distinct phase transitions occur within the measured temperature range. Similarly, below the melting point (408K) of the compound, the dielectric constant as a function of temperature also goes smoothly, and no dielectric anomaly is observed.

### Refinement

The C-bound H atoms were positioned geometrically, with C—H = 0.93-0.98 Å and refined as riding on their parent atoms with *U*_iso _(H) = 1.2U_eq_(C) or 1.5U_eq _(methyl). Atoms H2 and H1B were positioned geometrically and allowed to ride on N1, with N—H = 0.86 Å and *U*_iso _(H) = 1.2U_eq _(N).

### Crystal data

**Table:**

C_9_H_13_N_2_O^+^·ClO_4_^−^	*Z* = 2
*M**_r_* = 264.66	*F*(000) = 276
Triclinic, *P*1	*D*_x_ = 1.477 Mg m^−^^3^
Hall symbol: -P 1	Melting point: 408 K
*a* = 4.9342 (3) Å	Mo *K*α radiation, λ = 0.71073 Å
*b* = 8.973 (4) Å	Cell parameters from 1631 reflections
*c* = 13.715 (10) Å	θ = 2.3–27.4°
α = 93.046 (12)°	µ = 0.33 mm^−^^1^
β = 91.07 (2)°	*T* = 298 K
γ = 101.01 (3)°	Prism, colourless
*V* = 594.9 (5) Å^3^	0.2 × 0.2 × 0.2 mm

### Data collection

**Table:**

Rigaku SCXmini diffractometer	2708 independent reflections
Radiation source: fine-focus sealed tube	2160 reflections with *I* > 2σ(*I*)
graphite	*R*_int_ = 0.024
Detector resolution: 13.6612 pixels mm^-1^	θ_max_ = 27.5°, θ_min_ = 2.3°
ω scans	*h* = −6→6
Absorption correction: multi-scan (*CrystalClear*; Rigaku, 2005)	*k* = −11→11
*T*_min_ = 0.887, *T*_max_ = 1.000	*l* = −17→17
6065 measured reflections	

### Refinement

**Table:**

Refinement on *F*^2^	Primary atom site location: structure-invariant direct methods
Least-squares matrix: full	Secondary atom site location: difference Fourier map
*R*[*F*^2^ > 2σ(*F*^2^)] = 0.055	Hydrogen site location: inferred from neighbouring sites
*wR*(*F*^2^) = 0.159	H-atom parameters constrained
*S* = 1.07	*w* = 1/[σ^2^(*F*_o_^2^) + (0.078*P*)^2^ + 0.3515*P*] where *P* = (*F*_o_^2^ + 2*F*_c_^2^)/3
2708 reflections	(Δ/σ)_max_ = 0.001
154 parameters	Δρ_max_ = 0.78 e Å^−^^3^
0 restraints	Δρ_min_ = −0.58 e Å^−^^3^

### Special details

**Table:**

Geometry. All esds (except the esd in the dihedral angle between two l.s. planes) are estimated using the full covariance matrix. The cell esds are taken into account individually in the estimation of esds in distances, angles and torsion angles; correlations between esds in cell parameters are only used when they are defined by crystal symmetry. An approximate (isotropic) treatment of cell esds is used for estimating esds involving l.s. planes.
Refinement. Refinement of F^2^ against ALL reflections. The weighted R-factor wR and goodness of fit S are based on F^2^, conventional R-factors R are based on F, with F set to zero for negative F^2^. The threshold expression of F^2^ > 2sigma(F^2^) is used only for calculating R-factors(gt) etc. and is not relevant to the choice of reflections for refinement. R-factors based on F^2^ are statistically about twice as large as those based on F, and R- factors based on ALL data will be even larger.

### Fractional atomic coordinates and isotropic or equivalent isotropic displacement parameters (Å^2^)

**Table:**

	*x*	*y*	*z*	*U*_iso_*/*U*_eq_	
C1	0.8695 (6)	0.8140 (3)	0.8363 (2)	0.0502 (7)	
H1A	0.8291	0.9111	0.8408	0.060*	
C2	0.7491 (5)	0.7102 (3)	0.76352 (19)	0.0428 (6)	
H2A	0.6249	0.7363	0.7187	0.051*	
C3	0.8135 (4)	0.5660 (2)	0.75713 (17)	0.0329 (5)	
C4	0.9972 (5)	0.5295 (3)	0.82555 (19)	0.0398 (5)	
H4A	1.0437	0.4337	0.8223	0.048*	
C5	1.1098 (5)	0.6358 (3)	0.8980 (2)	0.0463 (6)	
H5A	1.2303	0.6120	0.9451	0.056*	
C6	0.6751 (5)	0.4515 (3)	0.67827 (17)	0.0340 (5)	
C7	0.7297 (5)	0.2295 (3)	0.5718 (2)	0.0452 (6)	
H7A	0.5280	0.2151	0.5658	0.054*	
C8	0.8048 (8)	0.0860 (3)	0.6075 (2)	0.0616 (8)	
H8A	0.7233	0.0652	0.6695	0.092*	
H8B	1.0020	0.0989	0.6144	0.092*	
H8C	0.7368	0.0026	0.5612	0.092*	
C9	0.8498 (9)	0.2688 (4)	0.4730 (2)	0.0703 (10)	
H9A	0.7961	0.3603	0.4532	0.105*	
H9B	0.7815	0.1871	0.4256	0.105*	
H9C	1.0476	0.2840	0.4779	0.105*	
Cl1	0.42620 (12)	0.18228 (8)	0.88680 (5)	0.0452 (2)	
N1	1.0451 (5)	0.7740 (3)	0.90049 (17)	0.0485 (6)	
H1B	1.1202	0.8401	0.9455	0.058*	
N2	0.8294 (4)	0.3550 (2)	0.64449 (17)	0.0436 (5)	
H2B	0.9970	0.3667	0.6664	0.052*	
O1	0.4371 (3)	0.4521 (2)	0.65134 (14)	0.0471 (5)	
O2	0.7111 (4)	0.1708 (2)	0.89323 (17)	0.0577 (5)	
O3	0.3117 (6)	0.1454 (5)	0.79332 (19)	0.1241 (15)	
O4	0.4067 (6)	0.3375 (3)	0.9161 (3)	0.0938 (10)	
O5	0.2732 (4)	0.0877 (2)	0.95541 (15)	0.0526 (5)	

### Atomic displacement parameters (Å^2^)

**Table:**

	*U*^11^	*U*^22^	*U*^33^	*U*^12^	*U*^13^	*U*^23^
C1	0.0574 (17)	0.0369 (13)	0.0581 (17)	0.0166 (12)	−0.0001 (13)	−0.0063 (12)
C2	0.0470 (14)	0.0389 (13)	0.0455 (14)	0.0165 (11)	−0.0033 (11)	0.0007 (10)
C3	0.0292 (10)	0.0301 (11)	0.0395 (12)	0.0063 (8)	0.0031 (9)	0.0008 (9)
C4	0.0389 (12)	0.0359 (12)	0.0459 (13)	0.0116 (10)	−0.0041 (10)	−0.0010 (10)
C5	0.0434 (14)	0.0511 (15)	0.0447 (14)	0.0123 (12)	−0.0060 (11)	−0.0022 (12)
C6	0.0290 (10)	0.0332 (11)	0.0394 (12)	0.0055 (9)	−0.0014 (9)	0.0010 (9)
C7	0.0358 (12)	0.0429 (14)	0.0553 (16)	0.0094 (10)	−0.0100 (11)	−0.0155 (12)
C8	0.085 (2)	0.0424 (15)	0.0561 (18)	0.0112 (15)	0.0005 (16)	−0.0060 (13)
C9	0.101 (3)	0.0553 (18)	0.0536 (18)	0.0164 (18)	−0.0149 (17)	−0.0046 (14)
Cl1	0.0334 (3)	0.0543 (4)	0.0474 (4)	0.0041 (3)	−0.0046 (2)	0.0162 (3)
N1	0.0520 (13)	0.0465 (13)	0.0451 (12)	0.0094 (10)	−0.0022 (10)	−0.0141 (10)
N2	0.0279 (10)	0.0429 (11)	0.0583 (13)	0.0093 (8)	−0.0097 (9)	−0.0169 (10)
O1	0.0309 (9)	0.0515 (11)	0.0599 (12)	0.0135 (8)	−0.0094 (8)	−0.0047 (9)
O2	0.0352 (10)	0.0632 (13)	0.0764 (14)	0.0129 (9)	0.0019 (9)	0.0071 (10)
O3	0.0714 (18)	0.243 (4)	0.0419 (14)	−0.011 (2)	−0.0133 (12)	0.0139 (19)
O4	0.0700 (16)	0.0436 (12)	0.174 (3)	0.0203 (11)	−0.0057 (17)	0.0286 (15)
O5	0.0568 (12)	0.0467 (11)	0.0558 (12)	0.0098 (9)	0.0161 (9)	0.0115 (9)

### Geometric parameters (Å, °)

**Table:**

C1—N1	1.333 (4)	C7—C9	1.521 (5)
C1—C2	1.373 (4)	C7—H7A	0.9800
C1—H1A	0.9300	C8—H8A	0.9600
C2—C3	1.388 (3)	C8—H8B	0.9600
C2—H2A	0.9300	C8—H8C	0.9600
C3—C4	1.387 (3)	C9—H9A	0.9600
C3—C6	1.509 (3)	C9—H9B	0.9600
C4—C5	1.372 (4)	C9—H9C	0.9600
C4—H4A	0.9300	Cl1—O3	1.389 (3)
C5—N1	1.337 (3)	Cl1—O5	1.429 (2)
C5—H5A	0.9300	Cl1—O2	1.4305 (19)
C6—O1	1.226 (3)	Cl1—O4	1.450 (3)
C6—N2	1.329 (3)	N1—H1B	0.8600
C7—N2	1.468 (3)	N2—H2B	0.8600
C7—C8	1.509 (4)		
			
N1—C1—C2	119.3 (2)	C7—C8—H8A	109.5
N1—C1—H1A	120.3	C7—C8—H8B	109.5
C2—C1—H1A	120.3	H8A—C8—H8B	109.5
C1—C2—C3	119.7 (2)	C7—C8—H8C	109.5
C1—C2—H2A	120.1	H8A—C8—H8C	109.5
C3—C2—H2A	120.1	H8B—C8—H8C	109.5
C4—C3—C2	119.0 (2)	C7—C9—H9A	109.5
C4—C3—C6	121.7 (2)	C7—C9—H9B	109.5
C2—C3—C6	119.3 (2)	H9A—C9—H9B	109.5
C5—C4—C3	119.5 (2)	C7—C9—H9C	109.5
C5—C4—H4A	120.3	H9A—C9—H9C	109.5
C3—C4—H4A	120.3	H9B—C9—H9C	109.5
N1—C5—C4	119.5 (2)	O3—Cl1—O5	110.28 (18)
N1—C5—H5A	120.2	O3—Cl1—O2	112.70 (17)
C4—C5—H5A	120.2	O5—Cl1—O2	109.78 (13)
O1—C6—N2	125.5 (2)	O3—Cl1—O4	109.5 (2)
O1—C6—C3	119.7 (2)	O5—Cl1—O4	106.56 (16)
N2—C6—C3	114.83 (19)	O2—Cl1—O4	107.79 (14)
N2—C7—C8	108.7 (2)	C1—N1—C5	123.0 (2)
N2—C7—C9	109.9 (2)	C1—N1—H1B	118.5
C8—C7—C9	112.4 (2)	C5—N1—H1B	118.5
N2—C7—H7A	108.6	C6—N2—C7	123.7 (2)
C8—C7—H7A	108.6	C6—N2—H2B	118.2
C9—C7—H7A	108.6	C7—N2—H2B	118.2
			
N1—C1—C2—C3	−0.6 (4)	C4—C3—C6—N2	−34.6 (3)
C1—C2—C3—C4	0.7 (4)	C2—C3—C6—N2	147.5 (2)
C1—C2—C3—C6	178.6 (2)	C2—C1—N1—C5	−0.4 (4)
C2—C3—C4—C5	0.3 (4)	C4—C5—N1—C1	1.4 (4)
C6—C3—C4—C5	−177.6 (2)	O1—C6—N2—C7	−2.7 (4)
C3—C4—C5—N1	−1.3 (4)	C3—C6—N2—C7	176.5 (2)
C4—C3—C6—O1	144.7 (2)	C8—C7—N2—C6	−131.6 (3)
C2—C3—C6—O1	−33.2 (3)	C9—C7—N2—C6	105.0 (3)

### Hydrogen-bond geometry (Å, °)

**Table:**

*D*—H···*A*	*D*—H	H···*A*	*D*···*A*	*D*—H···*A*
N1—H1B···O5^i^	0.86	2.20	2.879 (3)	136
N1—H1B···O2^ii^	0.86	2.36	3.032 (4)	135
N1—H1B···O5^iii^	0.86	2.55	2.918 (3)	107
N2—H2B···O1^iv^	0.86	2.18	2.957 (3)	150
C1—H1A···O2^v^	0.93	2.58	3.491 (4)	168
C4—H4A···O4^iv^	0.93	2.50	3.171 (3)	130
C5—H5A···O4^ii^	0.93	2.55	3.426 (4)	157
C7—H7A···O1	0.98	2.49	2.863 (3)	102

Symmetry codes: (i) *x*+1, *y*+1, *z*; (ii) −*x*+2, −*y*+1, −*z*+2; (iii) −*x*+1, −*y*+1, −*z*+2; (iv) *x*+1, *y*, *z*; (v) *x*, *y*+1, *z*.
